# Diagnosis and Assessment of Alcohol Use Disorders Among Adolescents

**Published:** 1998

**Authors:** Christopher S. Martin, Ken C. Winters

**Affiliations:** Christopher S. Martin, Ph.D., is assistant professor of psychiatry at the University of Pittsburgh School of Medicine and an investigator in the Pittsburgh Adolescent Alcohol Research Center, Pittsburgh, Pennsylvania. Ken C. Winters, Ph.D., is associate professor of psychiatry at the University of Minnesota School of Medicine, Minneapolis, Minnesota

**Keywords:** AODD (alcohol and other drug use disorder), adolescent, diagnostic criteria, diagnosis, patient assessment, validity (research methods), psychodiagnostic interview, identification and screening for AODD, symptom, disorder classification, self report, epidemiology, clinical study, treatment research, literature review

## Abstract

The diagnostic criteria for alcohol use disorders (AUDs) (i.e., alcohol abuse and alcohol dependence) as defined in the Diagnostic and Statistical Manual of Mental Disorders, Fourth Edition (DSM–IV) were developed largely from research and clinical experience with adults. Little is known about the validity of these criteria when applied to adolescents. Recent epidemiological and clinical studies of AUDs and their symptoms among adolescents have indicated that the DSM–IV criteria have significant limitations when applied to this age group. Diagnostic interviews and screening tools for adolescent AUDs are discussed. Numerous instruments are available that have shown moderate-to-high reliability and validity in assessing AUDs among adolescents.

Adolescent alcohol problems are an important public health issue. Research has indicated an increasing prevalence of adolescent alcohol use disorders (AUDs) (i.e., alcohol abuse and alcohol dependence) over recent decades. Approximately 40 percent of people with an AUD developed their first symptoms between the ages of 15 and 19 (Helzer et al. 1991). People with an earlier age of onset of AUDs tend to experience more severe alcohol problems and are more likely to have other psychiatric disorders (e.g., Babor et al. 1992). At the same time, longitudinal research has shown that drinking status and the presence of alcohol-related problems can change considerably across adolescence and into young adulthood. Much remains to be learned about the nature and development of alcohol problems during the teenage years.

The diagnostic criteria for AUDs have largely been developed based on research and clinical experience with adults. This article summarizes the role of diagnostic classification in the treatment and research of AUDs and describes the current diagnostic criteria for AUDs as defined in the *Diagnostic and Statistical Manual of Mental Disorders, Fourth Edition* (DSM–IV) (American Psychiatric Association 1994). Next, the article reviews epi-demiological and clinical research on DSM–IV AUD criteria among adolescents and potential limitations of these criteria when applied to this age group. Finally, the article describes some of the diagnostic interviews and screening tools that can be used to assess AUDs among adolescents.

## The Diagnosis of AUDs Among Adolescents

For any type of medical or psychiatric disorder, a valid diagnostic system is necessary to advance both treatment and research. Psychiatric disorders, including AUDs, are best viewed as evolving constructs that organize and describe a constellation of symptoms and behaviors. An accurate diagnostic system informs the clinician about course, prognosis, and the most effective treatment approaches. For researchers, diagnostic classification allows identification of subgroups and developmental pathways to the disorder. The standardized definitions provided by specific diagnostic criteria facilitate communication among and between researchers and clinicians. Although alcohol problems occur along a continuum of severity, specific diagnostic boundaries must be defined to guide both research and the allocation of limited health care resources.

### DSM–IV Diagnostic Criteria for AUDs

The DSM–IV describes two primary AUDs: alcohol abuse and alcohol dependence. A person receives a diagnosis of alcohol abuse if he or she experiences at least one of four abuse symptoms (i.e., role impairment, hazardous use, legal problems, and social problems) (see table below) that lead to “clinically significant impairment or distress.” These symptoms reflect either pathological patterns of alcohol use, psychosocial consequences, or both.

The framework for the diagnosis of alcohol dependence in the DSM–IV was influenced by the concept of the Alcohol Dependence Syndrome (ADS) developed by Edwards and Gross (1976). In the ADS, alcohol dependence is defined rather broadly—that is, as a constellation of symptoms related to physical dependence as well as compulsive and pathological patterns of alcohol use. To qualify for a DSM–IV diagnosis of alcohol dependence, a person must exhibit within a 12-month period at least three of the following seven dependence symptoms: (1) tolerance, (2) withdrawal or drinking to avoid or relieve withdrawal, (3) drinking larger amounts or for a longer period than intended, (4) unsuccessful attempts or a repeated desire to quit or to cut down on drinking, (5) much time spent using alcohol, (6) reduced social or recreational activities in favor of alcohol use, and (7) continued alcohol use despite psychological or physical problems (see table, p. 96). No single criterion is necessary or sufficient for an alcohol dependence diagnosis. Alcohol dependence is subtyped in DSM–IV as with or without physiological features, defined by tolerance or withdrawal symptoms.

**Table:** Symptoms of Alcohol Abuse and Alcohol Dependence as Defined in the *Diagnostic and Statistical Manual of Mental Disorders, Fourth Edition* (DSM–IV)

Alcohol Use Disorder	Brief Identifier of Symptom	Abstracted DSM–IV Definition
Alcohol Abuse	Role impairment	Frequent intoxication leading to failure to fulfill major role obligations (e.g., at school, work, or home)
	Hazardous use	Recurrent use when it is physically hazardous (e.g., driving while intoxicated)
	Legal problems	Recurrent alcohol-related legal problems
	Social problems	Continued drinking despite knowledge of persistent or recurrent social or interpersonal problems caused or exacerbated by alcohol use
Alcohol Dependence	Tolerance	Need to increase consumption by 50 percent or more to achieve the same effects; markedly reduced effects when drinking the same amount
	Withdrawal	Signs of alcohol withdrawal; drinking to avoid or relieve withdrawal
	Using more or longer than intended	Recurrent drinking of larger amounts or for a longer period of time than intended
	Quit/cut down	Unsuccessful attempts or a persistent desire to quit or cut down on drinking
	Much time spent using alcohol	Much time spent using, obtaining, or recovering from the effects of alcohol
	Reduced activities	Important social or recreational activities given up or reduced in favor of alcohol use
	Psychological/physical problems	Continued drinking despite knowledge of a recurrent or persistent psychological or physical problem caused or exacerbated by alcohol use

In contrast to previous versions of the DSM, the symptoms of alcohol abuse and alcohol dependence are mutually exclusive in DSM–IV. Moreover, the diagnoses of alcohol abuse and alcohol dependence are arranged hierarchically, such that a dependence diagnosis precludes an abuse diagnosis. Although not stated explicitly in DSM–IV, this hierarchical design implies that compared with alcohol dependence, alcohol abuse should be relatively mild and should onset at an earlier age.

The DSM–IV diagnostic criteria for AUDs are similar to the DSM–IV criteria for other drug use disorders (although some important differences do exist). Although this article focuses on adolescent AUDs, many of the diagnostic and assessment issues that are discussed apply to other drug use disorders as well. Because adolescent drinking and AUDs are strongly associated with other drug use and drug use disorders (e.g., Martin et al. 1996*a*), both alcohol and other drug use behaviors should be assessed in research and clinical settings.

## Studies of DSM–IV AUDs Among Adolescents

Several recent epidemiological and clinical studies have assessed DSM–IV AUD symptoms and diagnoses among adolescents. Epidemiological studies are important, because they provide estimates of the rates of symptoms and diagnoses in the general adolescent population. Clinical studies are equally important and complement epidemiological research by characterizing symptom patterns among adolescents who present for addiction treatment. Furthermore, clinical studies often provide detailed assessment of a large number of subjects with symptoms and diagnoses.

### Findings of Epidemiological Studies

Not surprisingly, the prevalence rates of adolescent AUDs vary according to age and gender. For example, Cohen and colleagues (1993) examined age-and gender-specific prevalences of AUDs in a representative household sample of 776 youth ages 10 to 20 in New York State. That study, which used DSM–III–R criteria, found that AUD prevalences jumped from 3.5 percent at ages 14 to 16 (3.1 percent of girls and 4.1 percent of boys) to 14.6 percent at ages 17 to 20 (8.9 percent of girls and 20.3 percent of boys). The observation that the prevalence of AUDs is higher in boys than in girls, particularly during late adolescence, has been confirmed in several studies.

Several recent studies have assessed DSM–IV alcohol symptoms and diagnoses in general population samples of adolescents. Lewinsohn and coworkers (1996) assessed DSM–IV AUD symptoms and diagnoses by interviewing a representative sample of 1,507 students ages 14 to 18 from urban and rural high schools in Oregon. Approximately 23 percent of the respondents had experienced at least one DSM–IV alcohol abuse or dependence symptom during their lifetime. The most common symptoms were dependence symptoms (i.e., tolerance, drinking larger amounts or for a longer period of time than intended, and reduced activities in favor of alcohol use) rather than abuse symptoms. The dependence symptoms of withdrawal and alcohol-related medical problems, and the abuse symptom of alcohol-related legal problems, were relatively rare. AUD diagnoses occurred in 6.2 percent of the sample at some time in their lives (1.9 percent of the sample had alcohol abuse and 4.3 percent had alcohol dependence). Another 16.7 percent of the sample had experienced some alcohol-related problems. This included 13.5 percent of participants who met the criteria for one or two DSM–IV dependence symptoms and no abuse symptoms, who therefore did not fulfill the DSM–IV criteria for an AUD.

Another large epidemiological study of alcohol and other drug use disorders assessed 74,008 9th and 12th grade high school students in Minnesota (Harrison et al. 1998). The lifetime presence of DSM–IV symptoms (all except withdrawal) was assessed by questionnaire; subjects were asked to respond affirmatively to questions about symptoms if they applied to either alcohol or other drugs. Among the 9th graders who had ever used alcohol or other drugs (approximately one-half of all 9th graders), 13.8 percent met the criteria for drug abuse and 8.2 percent met the criteria for drug dependence. Another 13 percent had one or two dependence symptoms and no abuse symptoms. The most common symptoms among the 9th graders were dependence symptoms: tolerance, using alcohol or drugs in greater amounts or for a longer time than intended, and much time spent using.

Approximately two-thirds of the 12th graders in the study had ever used alcohol or other drugs. Of those, 22.7 percent met the criteria for alcohol or other drug abuse, 10.5 percent met the criteria for alcohol or other drug dependence, and an additional 9.9 percent had one or two dependence symptoms and no abuse symptoms. The most common symptom in 12th graders was the abuse symptom of hazardous use (e.g., driving while intoxicated), followed closely by the dependence symptoms of tolerance, using alcohol or drugs in greater amounts or for a longer time than intended, and much time spent using, as well as the abuse symptom of social problems. Conversely, symptoms of alcohol-related medical and legal problems were rare. However, because questions about symptoms were not asked separately for alcohol versus other drugs in this study, the rate of alcohol and drug use disorders probably was overestimated, because in some cases positive answers about different symptoms may have applied to different substances. Therefore, these results should be interpreted cautiously. In addition, this study reported the prevalence of alcohol and drug use disorders only for adolescents who had ever used alcohol or other drugs. Accordingly, the results are not directly comparable to prevalence estimates for the entire adolescent population.

### Findings of Clinical Studies

Several recent studies have evaluated DSM–IV criteria for AUDs among clinical samples of adolescents. Martin and colleagues (1995) used an adapted version of the Structured Clinical Interview for the DSM (Spitzer et al. 1987) to examine DSM–IV symptoms of AUDs among adolescents ages 13 to 21 who were recruited from both clinical and community sources. The most common symptoms were the dependence symptoms of tolerance, drinking in greater amounts or for a longer period of time than intended, and much time spent using alcohol, as well as the abuse symptom of continued use despite social problems. Conversely, the dependence symptoms of withdrawal and alcohol-related medical problems and the abuse symptoms of hazardous use and alcohol-related legal problems were uncommon. For example, only 23 percent of the adolescents diagnosed with alcohol dependence (and none of the subjects without alcohol dependence) had experienced alcohol withdrawal. The high rates of tolerance and the low rates of withdrawal in this study are consistent with the results of a clinical study of adolescents by Stewart and Brown (1995) that used DSM–III–R criteria. The study by Martin and colleagues (1995) also identified five domains of recurrent alcohol-related problems not contained in the DSM–IV that were highly prevalent among adolescents with AUDs. Those problems were blackouts, passing out, risky sexual behavior, craving, and an alcohol-related drop in school grades.

Another investigation focused on DSM–IV criteria for AUDs in a clinical sample of 772 adolescents ages 12 to 19 (Winters et al. in press). AUD symptoms and diagnoses were assessed using the Adolescent Diagnostic Interview (ADI). The most common AUD symptoms in this study were the dependence symptoms of drinking in greater amounts or over longer periods than intended, unsuccessful attempts or a repeated desire to quit or cut down on drinking, and much time spent using alcohol. In contrast, the prevalence of withdrawal and alcohol-related legal problems was relatively low. Unlike the studies by Stewart and Brown (1995) and Martin and colleagues (1995), this investigation detected relatively low rates of tolerance, possibly because the ADI criteria for tolerance may be more conservative than those of other instruments.

### Limitations of the DSM–IV Criteria for AUDs in Adolescents

In general, the DSM–IV criteria for AUDs have shown some validity in adolescents, in that groups classified as having alcohol dependence, alcohol abuse, and no diagnosis tend to differ on measures of alcohol use, other drug use, and independent measures of alcohol problem severity (Lewinsohn et al. 1996; Martin et al. 1995; Winters et al. in press). However, the available data also suggest potential limitations of the DSM–IV criteria for AUDs when applied to adolescents. Some of these limitations may apply to adults as well.

One potential limitation is that the DSM–IV criteria appear to include several symptoms that are not typically experienced by adolescent problem drinkers. Some symptoms have a very low prevalence, even in clinical samples, and thus may have only limited utility. Those symptoms include withdrawal and alcohol-related medical problems, which generally emerge only after years of heavy drinking. Other symptoms may have limited utility because they tend to occur only in particular subgroups of adolescents. For example, the alcohol abuse symptom of hazardous use, which is usually assigned due to driving while intoxicated, is rare in early adolescence and then increases after age 16, although presumably only in youths with access to automobiles. Langenbucher and Martin (1996) reported that among adolescents, the symptoms of hazardous use and alcohol-related legal problems were highly related to male gender, increased age, and symptoms of conduct disorder.

Another limitation is that some DSM–IV symptoms may have low specificity for adolescents—that is, their presence does not clearly distinguish among adolescents with different levels of drinking problems. For example, the development of some tolerance to alcohol’s effects is likely a normal developmental phenomenon that occurs in most adolescent drinkers. The DSM–IV criteria define tolerance, in part, as the need to increase consumption by 50 percent or more to achieve the same effects. Thus, a need to consume three drinks to produce the same effect previously produced by two drinks would qualify as “tolerance” according to DSM–IV. Such a change in consumption at these relatively moderate drinking levels, however, likely occurs in most adolescent drinkers. Martin and colleagues (1995) found that tolerance was highly prevalent in adolescent drinkers with and without AUDs, even though this symptom was assigned only in subjects who consumed an average of five or more standard drinks per drinking occasion. Although marked tolerance to alcohol is an important aspect of alcohol dependence, difficulty in specifying and measuring this phenomenon makes it a problematic symptom for adolescents.

Other limitations of the DSM–IV criteria are related to the alcohol abuse category. The one-symptom threshold for the DSM–IV diagnosis of alcohol abuse, combined with the broad range of problems covered by the abuse symptoms, produces a great deal of heterogeneity among persons in this diagnostic category. A related issue is the lack of an accepted conceptual definition of alcohol abuse (Langenbucher and Martin 1996). Furthermore, the mutually exclusive DSM–IV categories of alcohol abuse and alcohol dependence symptoms are not clearly distinguished either conceptually or empirically. Some of the abuse and some of the dependence symptoms measure impaired control over drinking in the face of negative consequences. Harrison and colleagues (1998) found that measures of sensitivity, specificity and predictive power did not support the diagnostic distinction between abuse and dependence symptoms. Other investigators, however, have found results more supportive of the DSM–IV’s categorization of alcohol abuse and alcohol dependence symptoms (Lewinsohn et al. 1996).

A similar limitation of the DSM–IV AUD criteria among adolescents involves sequencing in the age of onset of alcohol abuse and alcohol dependence symptoms. Because alcohol abuse is usually considered as a relatively mild category relative to alcohol dependence, the onset of abuse symptoms would be expected to precede the onset of dependence symptoms. A study of sequencing in the age of symptom onset among adolescents, however, did not support the DSM–IV system (Martin et al. 1996*b*). The results suggested that DSM–IV alcohol symptoms developed in three distinct stages among adolescents, with some dependence symptoms occurring before some abuse symptoms, as follows:

- The first stage was characterized by three dependence symptoms (i.e., tolerance, drinking larger amounts or for a longer period of time than intended, and much time spent using alcohol) and two abuse symptoms (i.e., role impairment and social problems).
- The second stage was characterized by three dependence symptoms (i.e., unsuccessful attempts or a persistent desire to quit or cut down on drinking, reduced activities because of alcohol use, and continued use despite physical or psychological problems) as well as two abuse symptoms (i.e., hazardous use and alcohol-related legal problems).
- The third stage, which had the longest time to symptom onset, was characterized by the dependence symptom of withdrawal.

Finally, another apparent limitation of the DSM–IV criteria for AUDs is the existence of “diagnostic orphans”—that is, persons who exhibit one or two alcohol dependence symptoms and no alcohol abuse symptoms, who therefore do not qualify for a DSM–IV AUD. (For more information on diagnostic orphans, see sidebar, p. 100.)

**Diagnostic Orphans: Adolescents With Alcohol Symptoms but Without a DSM–IV Alcohol Use Disorder**

The *Diagnostic and Statistical Manual of Mental Disorders, Fourth Edition* (DSM–IV) (American Psychiatric Association 1994) describes two alcohol use disorders (AUDs), alcohol abuse and alcohol dependence, whose symptoms do not overlap. DSM–IV defines alcohol abuse by the presence of at least one of four symptoms and alcohol dependence by the co-occurrence of at least three of seven symptoms within a 1-year period (see table, p. 96). Kaczynski and Martin (1995) coined the term “diagnostic orphans” to describe adolescents with one or two of the alcohol dependence symptoms and none of the alcohol abuse symptoms, who therefore do not qualify for either a DSM–IV alcohol abuse or alcohol dependence diagnosis.

Epidemiological studies suggest that a substantial portion of adolescents are diagnostic orphans. Lewinsohn and colleagues (1996) found that 13.5 percent of high school students were diagnostic orphans. Harrison and coworkers (1998) found that, among those students who had ever used alcohol or other drugs, 13 percent of 9th graders and 9.9 percent of 12th graders were diagnostic orphans. Diagnostic orphans also have been described in a representative household sample of adults (Hasin and Paykin 1998).

In a study of adolescents drawn from clinical and community sources, Pollock and Martin (in press) found that diagnostic orphans represented about 31 percent of regular drinkers (i.e., adolescents who drank at least once a month for at least 6 months) who did not qualify for a DSM–IV alcohol abuse or dependence diagnosis. Diagnostic orphans were equally common among male and female regular drinkers. The most common symptoms exhibited by this group were tolerance, drinking larger amounts or for a longer period of time than intended, much time spent using alcohol, and unsuccessful attempts or a persistent desire to quit or cut down on drinking. Diagnostic orphans reported levels of drinking and other drug use and rates of drug use disorders that were similar to those of adolescents with an alcohol abuse diagnosis and significantly higher than those of adolescent regular drinkers without any DSM–IV alcohol symptoms. These results do not support the distinction between those with alcohol abuse (who do have a DSM–IV AUD) and diagnostic orphans (who do not have a DSM–IV AUD).

It is possible to conclude that adolescent diagnostic orphans have “fallen through the cracks” of the DSM–IV system for AUDs. Alternatively, the results could be interpreted as indicating that the one-symptom threshold for the DSM–IV diagnosis of alcohol abuse is too liberal, and that some adolescents with alcohol abuse diagnoses should not be classified as having an AUD. More research is needed to address these issues. Adolescent diagnostic orphans likely are an important group for treatment and prevention efforts.

***—Christopher S. Martin and Ken C. Winters***

ReferencesAmerican Psychiatric AssociationDiagnostic and Statistical Manual of Mental Disorders, Fourth EditionWashington, DCthe Association1994HarrisonPAFulkersonJABeebeTJDSM–IV substance use disorder criteria for adolescents: A critical examination based on a statewide school surveyAmerican Journal of Psychiatry1554864921998954599310.1176/ajp.155.4.486HasinDPaykinADependence symptoms but no diagnosis: Diagnostic “orphans” in a community sampleDrug and Alcohol Dependence5019261998958926910.1016/s0376-8716(98)00007-6KaczynskiNAMartinCSDiagnostic Orphans: Adolescents with Clinical Alcohol Symptomatology Who Do Not Qualify for DSM–IV Abuse or Dependence DiagnosesPaper presented at the annual meeting of the Research Society on AlcoholismSteamboat Springs, COJune 1995LewinsohnPMRohdePSeeleyJRAlcohol consumption in high school adolescents: Frequency of use and dimensional structure of associated problemsAddiction913753901996886720010.1046/j.1360-0443.1996.9133757.xPollockNKMartinCSDiagnostic orphans: Adolescents with alcohol symptomatology who do not qualify for DSM–IV abuse or dependence diagnosesAmerican Journal of Psychiatryin press10.1176/ajp.156.6.89710360129

## The Assessment of AUDs Among Adolescents

Clinicians and researchers use various approaches to assess alcohol problems in adolescents. The comprehensiveness of the assessment depends upon the purposes of the evaluation. One approach is the use of brief screening instruments—most commonly self-report questionnaires—to determine the possible presence of alcohol problems. (For more information on screening instruments, see sidebar, pp. 102–103.) If an initial screening indicates the need for further assessment, clinicians and researchers can employ diagnostic interviews to assign AUDs and to measure the nature and severity of alcohol problems. While this article emphasizes alcohol, most of these screening instruments and diagnostic interviews assess consumption patterns, problems and/or diagnoses for both alcohol and other drugs.

**Screening Instruments for Adolescent Alcohol Use Disorders**

In contrast to diagnostic interviews, which serve to establish a diagnosis of an alcohol use disorder (AUD), the aim of screening tools is to identify the *possible* presence of an alcohol problem or AUD. Thus, screening tools are used to determine whether a more complete assessment of a person’s condition and treatment needs is appropriate. Screening tools are typically self-report questionnaires that employ scoring cutoffs. The use of screening tools requires caution. A score above the cutoff point does not necessarily indicate the presence of an AUD but merely suggests that a more detailed assessment should be performed. Similarly, a score below the cutoff point does not necessarily indicate the absence of an AUD, but merely suggests that this is likely.

The following sections summarize some of the available screening tools that have been used widely with adolescents. Some of these instruments assess both alcohol and other drug use and problems, whereas others are specific to alcohol.

***Screening Tools for Alcohol and Other Drug Use Disorders***

***Client Substance Index—Short (CSI–S).*** The CSI–S (Thomas 1990) was developed and evaluated as part of a larger drug abuse screening protocol through the National Center for Juvenile Justice. The instrument is a 15-item yes/no questionnaire that is designed to identify juveniles within the court system who need additional assessment for alcohol and other drug problems. The CSI–S has shown good reliability. Scores on the CSI–S are consistent with other measures of adolescent alcohol and other drug problems, and the instrument discriminates among adolescent groups defined according to the severity of their criminal offenses.

***Drug and Alcohol Problem (DAP) Quick Screen.*** This 30-item questionnaire has been tested in a pediatric practice setting (Schwartz and Wirtz 1990). Studies have indicated that these items measure overall alcohol and other drug problem severity. The reliability and validity of the DAP Quick Screen, however, have not been evaluated.

***Drug Use Screening Inventory—Revised (DUSI–R).*** The adolescent version of the DUSI–R (Tarter et al. 1992) assesses alcohol and other drug use patterns as well as psychosocial functioning in different life areas using 159 true/false questions. This tool, which was developed from the same initial pool of items as was the Problem Oriented Screening Instrument for Teenagers (described below), yields scores on 10 functional adolescent problem areas: alcohol and other drug use, physical health, mental health, family relations, peer relationships, educational status, vocational status, social skills, leisure and recreation, and aggressive behavior/delinquency. The DUSI–R also includes a lie scale and has lifetime, past-year, and past-month versions. The adolescent version of the DUSI–R has shown good reliability and validity (Kirisci et al. 1995). For example, the scores on certain DUSI–R subscales are related to alcohol and other drug use disorder diagnoses among adolescents.

***Perceived Benefit of Drinking and Drug Use.*** This 10-item questionnaire, which asks questions about the perceived benefits of alcohol and other drug use, was developed as a nonthreatening problem severity screen. It is based on the approach that beliefs about drug use, particularly the expected personal benefits of using alcohol and other drugs, tend to be associated with actual alcohol and other drug use. The validity of this instrument is supported by findings that in both school samples and adolescent psychiatric inpatient samples, test scores are related to other measures of alcohol and other drug use and associated problems (Petchers and Singer 1990).

***Personal Experience Screening Questionnaire (PESQ).*** The PESQ is a 40-item questionnaire that provides measures of overall problem severity, alcohol and other drug use history, certain psychosocial problems, and response-distortion tendencies (i.e., the tendency to exaggerate or minimize responses about alcohol and other drug use behaviors) (Winters 1992). Cutoff scores indicating the need for further assessment have been established and validated for normal adolescents, juvenile offenders, and adolescents in addiction treatment.

***Problem Oriented Screening Instrument for Teenagers (POSIT).*** This 139-item yes/no questionnaire is part of the Adolescent Assessment and Referral System developed by the National Institute on Drug Abuse (Rahdert 1991). The POSIT was developed from the same pool of initial items as the DUSI–R (described previously). It addresses 10 areas of adolescent functioning (e.g., alcohol and other drug use, mental health, family relations, educational status, and aggressive behavior/delinquency). Cutoff scores indicating the need for further assessment have been established. Several investigators have reported evidence supporting the validity of the POSIT (e.g., Dembo et al. 1997).

***Substance Abuse Subtle Screening Inventory (SASSI).*** Miller’s (1985) adolescent version of the SASSI consists of 81 questions pertaining to the severity of alcohol and other drug problems. The SASSI yields scores for alcohol problems, other drug problems, and defensiveness (i.e., the tendency to minimize or deny problems). Validity data indicate that the SASSI cutoff score suggesting “chemical dependency” corresponds highly with diagnoses of alcohol and other drug use disorders obtained upon treatment entry (Risberg et al. 1995).

***AUD-Specific Screening Tools***

***Adolescent Alcohol Involvement Scale (AAIS).*** The AAIS is a 14-item questionnaire that examines current and past alcohol consumption, drinking context, short-and long-term effects of drinking, and perceptions about drinking (Mayer and Filstead 1979). An overall score describes the degree of alcohol involvement. The AAIS scores are significantly related to AUD diagnoses, independent clinical assessments of severity, and parental reports. Cutoff scores have been established for 13- to 19-year-olds from both clinical and nonclinical samples.

***Adolescent Drinking Index (ADI).*** The ADI measures adolescent problem drinking using 24 items addressing alcohol problems related to psychological, physical and social functioning, as well as impaired control over drinking behavior. The instrument yields an overall severity score as well as two subscale scores reflecting self-medicating drinking and rebellious drinking. Studies have confirmed the reliability and validity of this tool. Scores on the ADI are associated with alcohol consumption levels and differ significantly among adolescents with different levels of alcohol problem severity (Harrell and Wirtz 1989).

***Rutgers Alcohol Problem Index (RAPI).*** The RAPI is a 23-item questionnaire that focuses on consequences of alcohol use pertaining to family life, social relations, psychological functioning, delinquency, physical problems, and neuropsychological functioning (White and Labouvie 1989). Positive responses to the RAPI questions were found to correlate with AUD diagnoses.

***—Christopher S. Martin and Ken C. Winters***

ReferencesDemboRSchmeidlerJBordenPChin SueCManningDUse of the POSIT among arrested youths entering a juvenile assessment center: A replication and updateJournal of Child and Adolescent Substance Abuse619421997HarrellAWirtzPMScreening for adolescent problem drinking: Validation of a multidimensional instrument for case identificationPsychological Assessment161631989KirisciLMezzichATarterRNorms and sensitivity of the adolescent version of the drug use screening inventoryAddictive Behaviors201491571995748430910.1016/0306-4603(94)00058-1MayerJFilsteadWJThe Adolescent Alcohol Involvement Scale: An instrument for measuring adolescent use and misuse of alcoholJournal of Studies on Alcohol40291300197944934810.15288/jsa.1979.40.291MillerGThe Substance Abuse Subtle Screening Inventory—Adolescent VersionBloomington, INSASSI Institute1985PetchersMSingerMClinical applicability of a substance abuse screening instrumentJournal of Adolescent Chemical Dependency147561990RahdertEThe Adolescent Assessment/Referral System ManualDHHS Pub. No. (ADM) 91–1735Rockville, MDU.S. Department of Health and Human Services, National Institute on Drug Abuse1991RisbergRAStevensMJGraybillDFValidating the adolescent form of the Substance Abuse Subtle Screening InventoryJournal of Child and Adolescent Substance Abuse425411995SchwartzRHWirtzPWPotential substance abuse: Detection among adolescent patients using the Drug and Alcohol Problem (DAP) Quick Screen, a 30-item questionnaireClinical Pediatrics2938431990240350010.1177/000992289002900106TarterRELairdSBBuksteinOKaminerYValidation of the adolescent drug use screening inventory: Preliminary findingsPsychology of Addictive Behaviors63223261992ThomasDWSubstance Abuse Screening Protocol for the Juvenile CourtsPittsburghNational Center for Juvenile Justice1990WhiteHRLabouvieEWTowards the assessment of adolescent problem drinkingJournal of Studies on Alcohol5030371989292712010.15288/jsa.1989.50.30WintersKCDevelopment of an adolescent alcohol and other drug abuse screening scale: Personal Experience Screening QuestionnaireAddictive Behaviors174794901992133243410.1016/0306-4603(92)90008-j

### Self-Reports and Their Validity

Self-reports provide the most direct information about a person’s alcohol and other drug use and associated problems, which is often not available from any other source. As such, self-reports are critical for diagnostic assessment. The validity of self-reported alcohol and other drug use behaviors, however, has been the subject of considerable debate. In addition to purposely distorting the truth, clients may provide inaccurate responses because of lack of insight, inattentiveness, or misunderstanding of a question. Furthermore, adolescents with alcohol and other drug problems are sometimes developmentally delayed in terms of their cognitive, social, and emotional functioning, which may affect their perception of problems and their willingness to report them. However, the literature does provide some support for the validity of adolescent self-reports of alcohol and other drug use and related problems (Maisto et al. 1995), as follows:

- A large proportion of youth in addiction treatment settings admit to the use of alcohol and other drugs and associated problems.
- Few adolescents in treatment endorse questions that indicate the faking of responses (e.g., high scores on “lie” scales of questionnaires or admitting to the use of a fictitious drug).
- The information provided by adolescents is usually in general agreement with information obtained from other sources (e.g., parents, peers, and archival records).
- The information provided in adolescent self-reports generally remains consistent over time.

Nevertheless, inconsistent self-reports have been noted in the literature. When adolescents were asked about infrequent past alcohol and drug use and when queried over a 1-year interval about the age of initial alcohol and other drug use, significant inconsistencies have been observed (e.g., Single et al. 1975). Furthermore, clinical experience suggests that many adolescents entering treatment tend to minimize the extent of their alcohol and other drug use and the severity of associated problems. In fact, some investigators have observed that adolescents sometimes report greater past alcohol and other drug use and related problems at treatment completion than at treatment entry (e.g., Stinchfield 1997).

The complex issues regarding the validity of self-reports warrant further research. Researchers should consider the effects of how the information is gathered on the degree of self-disclosure. Several studies have indicated that questionnaires administered by computer and pencil-and-paper methods tend to yield equivalent responses concerning alcohol and other drug use behaviors. Some research with adults has shown slightly higher reports of alcohol and other drug use when the information is obtained through questionnaires as opposed to interviews. Similar studies have not yet been conducted with adolescents.

### Parent Reports and Their Validity

Another commonly used information source regarding adolescent alcohol and other drug use and associated problems are the youths’ parents. Clinical experience has long suggested, however, that many parents cannot provide meaningful details about their child’s alcohol and other drug use behaviors. Studies on this topic have yielded inconsistent results. In studies comparing diagnoses of alcohol and other drug use disorders based on parent reports with those based on self-reports, diagnostic agreement has ranged from 17 percent (Weissman et al. 1987) to 63 percent (Edelbrock et al. 1986). Another recent study of adolescents in addiction treatment compared self-reports and mother reports regarding a wide range of alcohol and other drug use behaviors (Winters et al. 1996). In that study, the concurrence of self-reports and mother reports of alcohol and drug use and related consequences was modest. The findings showed that most often the mother under-reported alcohol and drug use behaviors compared to the adolescent’s report.

### Diagnostic Interviews

Diagnostic interviews, in which clients are asked a set of predetermined questions, are considered by many researchers and clinicians to be the most comprehensive measures of alcohol and other drug use disorders. With the advent of definable diagnostic criteria for these disorders, such as those delineated in DSM–IV, diagnostic interviews can more precisely and reliably elicit the information needed to make a diagnosis. Furthermore, diagnostic interviews use standardized symptom definitions and question formats, which help minimize variability in responses. The use of followup questions provides important information that cannot always be obtained through the more rigid format of a questionnaire.

Diagnostic interviews are often described as either structured or semi-structured, based on the way in which they are administered and the degree of clinical judgment that the interviewer must employ when asking questions and when assigning symptoms and diagnoses. Highly structured interviews direct the interviewer to read verbatim a series of questions in a decision-tree format. In the decision-tree format, different responses lead to specific followup questions that assess the nature, persistence, duration, and clinical impact of alcohol- and drug-related problems. The interviewer rates each symptom as either absent or present, according to detailed written symptom definitions. Most structured interviews can be administered with acceptable reliability by a well-trained lay person. Semi-structured interviews require the interviewer to elicit an initial response and then determine, through additional unstructured probing, whether a symptom is present or absent. Such interviews allow considerable latitude in adapting questions to suit the respondent, and therefore usually require more advanced training in assessment. In terms of consistency of results across different interviewers, semi-structured interviews are often at a disadvantage compared with structured interviews, because they involve greater clinical judgment in scoring the responses. Many professionals believe, however, that this format can produce more comprehensive information than can fully structured interviews.

### Selecting Diagnostic Interviews for Adolescent AUDs

Given the considerable amount of expertise, time, and resources needed to develop sound diagnostic instruments, it is generally most cost-effective to use interviews whose properties have already been assessed. Many recent instruments have been extensively studied and have proven to be reliable and valid. Several handbooks and review articles are available that can guide the selection of an appropriate interview (e.g., Allen and Columbus 1995; McLellan et al. 1998).

A number of criteria should be considered when selecting a diagnostic interview for adolescent AUDs and other drug use disorders, including the following:

- The diagnostic interview should have demonstrated adequate measurement properties of reliability and validity. Reliability refers to the consistency of results across different interviewers or assessments. Validity refers to whether an interview measures what it is supposed to measure. Validity is often assessed by comparing interview results with other measures that are known to accurately assess diagnoses.
- Assessment should preferably involve both lifetime and recent (e.g., within the past year) time frames, because a lifetime perspective can provide important information about the course and chronicity of a disorder, while the profile of recent symptoms has obvious clinical, research, and diagnostic value.
- For positive responses, the instrument should contain questions related to the ages of both onset and offset of symptoms.
- The interview should provide questions to assess whether a problem was sufficiently persistent, recurrent, or clinically significant to warrant a positive symptom rating.
- Unless some of the information is provided by other assessment tools, the diagnostic interview should assess a wide variety of alcohol-and drug-related behaviors, including alcohol and other drug use, problems not contained in the diagnostic criteria, prior treatment experiences, family and peer alcohol and drug use, school functioning, and mental health status.
- The interview’s ease of administration, length, and training requirements must be compatible with the assessment goals. For example, researchers generally require interviews that efficiently yield reliable and detailed data. In contrast, some clinicians may be more interested in measures that are not excessively time consuming and require only modest training to administer.

## Commonly Used Diagnostic Interviews for Adolescents

A number of diagnostic interviews can be used to assess adolescent alcohol and other drug use disorders. Some of those instruments focus primarily on alcohol and other drug use disorders, whereas others are general psychiatric interviews that contain specific sections for assessing those disorders. The following sections summarize some of those diagnostic interviews. The list emphasizes interviews that have been adapted for DSM–IV criteria and are widely used in the United States.

### General Psychiatric Interviews

#### Diagnostic Interview for Children and Adolescents (DICA)

The DICA is a long-standing structured psychiatric interview. A revised version incorporating the DSM–IV criteria now exists (Reich et al. 1992). Although no studies have specifically evaluated the DICA’s measurement properties regarding AUDs, general findings indicate that this instrument is reasonably reliable and valid.

#### Diagnostic Interview Schedule for Children (DISC–C)

The structured DISC–C has undergone several adaptations, the most recent of which is based on the DSM–IV (Shaffer et al. 1996). A separate version of the interview exists for parents. Both the child and the parent versions of the DISC have shown good sensitivity in identifying youth who have received an independent medical diagnosis of an alcohol or drug use disorder (Fisher et al. 1993). However, the DISC–C has shown only modest reliability for DSM–III–R alcohol and other drug use disorders (Roberts et al. 1996).

#### Kiddie SADS (K–SADS)

This popular semi-structured interview, which is organized around the Research Diagnostic Criteria, is a child and adolescent version of the Schedule for Affective Disorders and Schizophrenia. Symptoms of alcohol and other drug use disorders are contained in the version of the interview that addresses lifetime symptoms (K–SADS–E) (Orvaschel 1985). A DSM–IV version now exists (K–SADS–E–5) (Orvaschel 1995). Reliability and validity studies of the K–SADS–E provide no data regarding alcohol and other drug use disorders, so the use of this interview among youth with alcohol and drug problems should proceed cautiously.

#### Structured Clinical Interview for the DSM (SCID)

The SCID is a structured interview developed to assesses psychiatric disorders according to DSM criteria in adults. The SCID provides specific operational definitions for each symptom and verbatim questions in a decision-tree format. The interviewer rates each symptom as absent, subclinical, or clinically present. The SCID is used widely, and the DSM–III–R section on alcohol and other drug use disorders has shown good reliability with adults. Martin and colleagues (1995) modified the DSM–III–R version of the drug use disorders section of the SCID (Spitzer et al. 1987) to assess DSM–IV alcohol and other drug use disorders among adolescents. Symptoms and diagnoses established with this version of the SCID have shown good concurrent validity (i.e., are associated with measures of drinking and problem severity assessed at the same time). In addition, preliminary analyses have suggested moderate to high agreement among interviewers (i.e., inter-rater reliability).

### Interviews Focusing on Alcohol and Other Drug Use Disorders

#### Adolescent Diagnostic Interview (ADI)

The ADI assesses the symptoms of alcohol and other drug use disorders as defined in both the DSM–III–R and DSM–IV. The ADI also measures sociodemographic information; alcohol and other drug use history; and psychosocial functioning, including mental health. The ADI’s reliability and validity are moderate to high (Winters and Henly 1993).

#### Customary Drinking and Drug Use Record (CDDR)

The CDDR is a structured interview that measures alcohol and other drug use for both recent (i.e., past 3 months) and lifetime periods, the presence of DSM–III–R and DSM–IV dependence symptoms for alcohol and other drug use disorders, and several negative consequences that are similar to DSM–III–R and DSM–IV alcohol and other drug abuse symptoms. The CDDR has high reliability across all major content domains and good concurrent validity. The CDDR has been found to discriminate between youth in the general population and those in treatment and produces results consistent with those of other diagnostic instruments (Brown et al. in press).

## Summary

Any diagnostic system applied to adolescent alcohol problems should reflect current knowledge of the nature and development of those problems. The diagnostic criteria for AUDs in the DSM–IV, however, were developed largely from research and clinical experience with adults. Although the number of studies is small, the available data suggest important limitations of the DSM–IV AUD criteria when applied to adolescents. More research is needed to evaluate potential changes in diagnostic criteria that may better represent the nature and development of adolescent alcohol problems. It is an open question whether future changes in diagnostic criteria for AUDs can provide a unified system that is equally valid for both adults and adolescents, or whether adolescent-specific clinical and research criteria for AUDs should be developed.

Research has generally supported the validity of self-reports of alcohol and other drug problems obtained from teenagers in clinical settings. Future research should identify characteristics of the individual adolescent and of the setting in which the information is obtained that influence the validity of self-reports. Clinicians and researchers have numerous options when selecting diagnostic instruments and screening measures for adolescent AUDs. Many of these instruments have favorable reliability and validity, and several reviews provide an overview to help guide the choice of an appropriate assessment instrument (e.g., Allen and Columbus 1995; McLellan et al. 1998).
